# Exploring stressor exposure and mental health symptom patterns among forcibly displaced people: A latent profile analysis

**DOI:** 10.1002/jts.70110

**Published:** 2026-07-28

**Authors:** Lisa Dasen, Jennifer Kurath, Béatrice Strock, Karl Juri Weinreich, Julia Spaaij, Matthis Schick, Angela Nickerson, Richard A. Bryant, Naser Morina

**Affiliations:** ^1^ Department of Consultation‐Liaison Psychiatry and Psychosomatic Medicine University Hospital Zurich University of Zurich Zurich Switzerland; ^2^ School of Psychology University of New South Wales Sydney Australia; ^3^ Black Dog Institute Sydney Australia

## Abstract

The association between trauma exposure and mental health among forcibly displaced people (FDP) is well‐documented. However, prior research has largely employed variable‐centered approaches, which may obscure heterogeneity in individual experiences. To date, no study has applied a person‐centered framework to examine patterns of stressor exposure and mental health outcomes in FDP. To address this gap, this study aimed to identify distinct subgroups of FDP who experience similar patterns of stressor exposure and mental health symptoms and examine predictors of subgroup membership. Participants were 437 FDP residing in Switzerland. Latent profile analysis was used to classify individuals into subgroups according to reported levels of stressor exposure and mental health outcomes. Sociodemographic and psychosocial predictors of profile membership were analyzed using multinomial logistic regression. Five distinct profiles emerged, characterized as: (a) very high stressor exposure, very high symptom levels (11.2%); (b) high stressor exposure, high symptom levels (43.0%); (c) moderate stressor exposure, moderate symptom levels (28.8%); (d) moderate stressor exposure, low symptom levels (11.9%); and (e) low stressor exposure, no symptoms (5.0%). Male gender, higher perceived social support, and secure visa status were significantly associated with membership in lower‐symptom profiles. The findings suggest that person‐centered approaches may offer valuable insights into the association between stressor exposure and mental health among FDP. The identified profiles indicate that these associations are not uniform, underscoring heterogeneity in stressor responses within this population. Such profiles help identify both vulnerable and resilient individuals and support tailoring interventions to individual profiles.

Forcibly displaced people (FDP) are commonly exposed to multiple, often chronic and cumulative stressors, including potentially traumatic experiences (PTEs) that occur before and during displacement, such as torture, loss of property, and the death of loved ones (Sigvardsdotter et al., 2016). In addition to these pre‐ and perimigration stressors, FDP often face ongoing postmigration challenges in their host countries, including discrimination, social isolation, and uncertainty regarding asylum and legal status (Gleeson et al., 2020; Hynie, 2018). Together, these stressors contribute to markedly elevated rates of mental disorders in FDP, particularly posttraumatic stress disorder (PTSD) and depression (Blackmore et al., 2020; Bryant, Nickerson, et al., 2023). Meta‐analytic evidence indicates that major depression is approximately 7 times more prevalent and PTSD 4–5 times more prevalent than in the general population (Koenen et al., 2017; Patanè et al., 2022), whereas the prevalence rates for anxiety are more comparable (Blackmore et al., 2020; Henkelmann et al., 2020).

Research examining these links between trauma exposure and mental health has primarily relied on variable‐centered approaches, which assume that associations between variables and outcomes are uniform across the entire population (Howard & Hoffman, 2018). This assumption is problematic, given that psychiatric disorders such as PTSD demonstrate substantial heterogeneity in symptom patterns and trajectories (Bryant, Galatzer‐Levy, & Hadzi‐Pavlovic, 2023; Galatzer‐Levy & Bryant, 2013). Consequently, variable‐centered approaches may, therefore, obscure meaningful individual differences in trauma responses.

Person‑centered approaches, including latent class analysis (LCA) and latent profile analysis (LPA), offer a means to disentangle this heterogeneity by identifying unobserved subgroups characterized by shared symptom patterns (Andersson et al., 2024). Whereas LCA uses dichotomous indicators, LPA relies on continuous variables (Bauer, 2022). These approaches can help to identify subgroups exhibiting subclinical psychopathology, comorbidity, or particularly vulnerable profiles. This is crucial for developing targeted clinical interventions and tailoring support strategies to enhance the effectiveness of mental health care for FDP.

In research on FDP, person‐centered approaches are increasingly used to capture heterogeneity in psychological symptom responses (Lenferink et al., 2022; Minihan et al., 2018; Nickerson, Hadzi‐Pavlovic, et al., 2019). For example, in a representative sample of refugees, Nickerson, Hadzi‐Pavlovic, et al. (2019) identified five symptom classes ranging from highly comorbid to resilient profiles, with the largest subgroup characterized by a resilient profile marked by low levels of psychological symptoms. These findings demonstrate that FDP exhibit diverse psychological symptom patterns and that only a subset of trauma‐exposed individuals develop clinically significant psychopathology, whereas others maintain relatively low symptom levels indicative of resilience (Bonanno et al., 2024).

Recent years have seen a growing research focus on resilience (Wolke et al., 2025). Although consensus regarding its definition and operationalization remains limited, resilience is increasingly conceptualized as a dynamic process of adaptation to adversity, commonly defined as maintaining or quickly returning to good mental health during and after adversity (Kalisch et al., 2021). Within this framework, resilience can be operationalized as individuals’ mental health status in relation to their cumulative stressor load, defined as the total number of stressors experienced within a given time frame. One widely used method to quantify resilience is the *residualization approach*, which captures individual deviations from the average response to adversity (Cahill et al., 2022; Kalisch et al., 2015, 2021). Accordingly, individuals with comparatively low psychological symptoms despite high stressor exposure are considered more resilient than those with similar exposure but poorer outcomes.

Accurately assessing stressor load (i.e., the sum of experienced stressors) requires considering a broad spectrum of stressors, including both primary stressors, such as torture and war, and ongoing postmigration stressors, such as legal uncertainty or social isolation (Kalisch et al., 2015, 2021). This is particularly important in refugee populations due to heterogeneity in the type, intensity, and timing of stressor exposure (Byrow et al., 2022; Nickerson et al., 2021). Although the residualization approach accounts for differences in stressor exposure and avoids conflating resilience with lower adversity (Kalisch et al., 2021), it is sensitive to outliers and assumes a largely continuous distribution of resilience, potentially obscuring qualitatively distinct subgroups, such as individuals with particularly low or high resilience (Höltge & Ungar, 2022).

Given this heterogeneity, person‐centered approaches may offer a promising framework for clarifying how different patterns of trauma exposure relate to different mental health outcomes among FDP (Luthar, 2015; Miller‐Lewis et al., 2013). Identifying subgroups that differ in both stressor exposure patterns and mental health profiles may provide a more nuanced understanding of intraindividual adaptation following forced displacement. Recent studies using LPA have identified distinct adaptation patterns, including resilient subgroups characterized by high adversity but low distress (de Maat et al., 2023; Yang et al., 2023). For example, Yang et al. (2023) identified four profiles (normative: 59.2%, vulnerable: 29.0%, resilient: 8.0%, adverse: 3.8%) in a sample of individuals exposed to bullying. To our knowledge, no study has yet applied a person‐centered framework to jointly examine patterns of stressor exposure and mental health outcomes in FDP.

In addition to examining different latent profiles, it is useful to identify factors associated with profile membership to better understand individual risk factors across subgroups. Sociodemographic factors, such as female gender and older age, have been associated with poorer mental health among FDP in high‐income countries (Nickerson et al., 2024). Moreover, higher levels of educational attainment may reduce the risk of developing mental disorders (Barbui et al., 2023), although it has also been linked to poorer outcomes, potentially due to status loss (Renner et al., 2021). Displacement‐related factors may further influence mental health. Longer residence in the host country (Schweitzer et al., 2006) and insecure visa status (Nickerson, Byrow, et al., 2019) have also been associated with poorer mental health and reduced integration (Schick et al., 2016). Finally, social support is one of the most consistently observed protective factors (Nickerson et al., 2024). For example, higher perceived social support was found to predict membership in resilient subgroups in a sample of internally displaced individuals (Heeke et al., 2017).

The present study applied LPA to identify patterns of stressor exposure and mental health outcomes in FDP and examined sociodemographic and psychosocial predictors of subgroup membership. Based on research by Fergus and Zimmerman (2005), we expected to observe at least four profiles reflecting combinations of stressor exposure (low vs. high) and mental health (good vs. poor). Specifically, we hypothesized that the sample would include (a) a high stressor exposure/low symptom group, (b) a high stressor exposure/high symptom group, (c) a low stressor exposure/low symptom group, and (d) a low stressor exposure/high symptom group. We further hypothesized that male gender, younger age, secure visa status, shorter length of residency, higher educational attainment, and higher perceived social support would be associated with membership in the high stressor exposure/low symptom (i.e., resilient) profile compared to those characterized by higher symptom levels.

## METHOD

### Participants and procedure

#### Recruitment and assessment

This cross‐sectional study was conducted within the Scaling‑Up Psychological Interventions in Refugees in Switzerland (SPIRIT) Project (n.d.) examining mental health outcomes and associated factors among FDP residing in Switzerland, as well as the effects of a brief psychological intervention, *Problem Management Plus* (PM+; Dawson et al., 2015), on mental health and social integration. Data were collected between April and December 2024. Participant recruitment was conducted in collaboration with local partner organizations (e.g., the cantonal Swiss Red Cross and Caritas) at PM+ implementation sites across Switzerland. Upon registration, local coordinators scheduled an initial mental health assessment. Assessments were administered by master's‐level psychology students proficient in German, French, or English or by trained research assistants fluent in the study languages. All assessors received comprehensive training in the administration of mental health instruments, psychological first aid, and good clinical practice (GCP), with weekly supervision from the first and second authors.

Assessments were conducted at locations convenient for participants (e.g., municipal offices, refugee centers, or central facilities). Participants received detailed study information and provided written informed consent prior to data collection; identical procedures applied to adults and minors (i.e., FDP aged 16–18 years). All instruments were administered digitally via the secure Unipark platform (Tivian, 2026) as self‐report measures, with sessions lasting approximately 45–60 min. Assessors clarified items when needed, and accredited interpreters were used if language barriers arose. Participants received no financial compensation but were offered free access to PM+. The study protocol was approved by the Ethics Committee of the canton of Zurich, Switzerland (BASEC‐Nr. 2023‐00857). The study was conducted in accordance with the current version of the Declaration of Helsinki and all applicable local legal and regulatory requirements. All participants signed an informed consent form to participate in the study.

#### Inclusion and exclusion criteria

Participants were a culturally and linguistically diverse group of FDP living in Switzerland who had registered for PM+. Inclusion criteria for the study were (a) ongoing asylum process or hold a recognized refugee status; (b) aged 16 years or older; and (c) ability to speak one of the 12 study languages sufficiently (i.e., Arabic, English, Farsi/Dari, French, Kurdish Kurmanji, Pashto, Russian, Tamil, Tigrinya, Turkish, Ukrainian, or German). Individuals with severe cognitive impairments, psychotic or dissociative symptoms, and acute suicidality were excluded.

### Measures

All measures were administered in the 12 study languages. Validated translations were used where available; otherwise, German and French versions were generated from English using the DeepL translation tool (Deepl, n.d.) and subsequently proofread by native‐speaking members of the research team. For all other languages, measures were translated from English by accredited translators and proofread, with additional review by native‑speaking research assistants to ensure linguistic accuracy.

#### Stressor exposure

To accurately assess individual stressor exposure, a broad spectrum of potential stressors was considered. This included both PTEs (e.g., trauma or war) and ongoing postmigration stressors (e.g., legal uncertainty or social isolation). A comprehensive overview of all assessed PTEs and postmigration stressors is available in Supplementary Table S1.

##### PTEs

PTE exposure was assessed using a checklist of seven event types, capturing whether participants had experienced or witnessed each type (O'Donnell et al., 2020). A cumulative trauma exposure score was computed (range: 0–7).

##### Postmigration stressors

Postmigration stressors were assessed using an adapted version of the Post‐Migration Living Difficulties Checklist (PMLDC; Schweitzer et al., 2018). The measure included 10 items, with Items 9 and 10 (“loneliness, boredom, or isolation”) merged in line with previous adaptations for the Swiss context (Schick et al., 2016). Items were rated on a 5‐point scale ranging from 0 (*not a problem/did not occur*) to 4 (*a very serious problem*) and dichotomized, with ratings of 2 or higher indicating the presence of a stressor. An overall score was calculated by summing all endorsed items (range: 0–9). Adapted versions of the PMLDC are well established in refugee research (Carswell et al., 2011; Schweitzer et al., 2006).

#### Mental health outcomes

Two self‐report questionnaires were used as indicators of current mental health. Consistent with a multidimensional, transdiagnostic framework (Dalgleish et al., 2020), these questionnaires assessed a broad range of symptom domains, allowing for a more comprehensive evaluation of mental health.

##### Depression and anxiety

Symptoms of depression and anxiety were assessed using the Hopkins Symptom Checklist (HSCL‐25; Derogatis et al., 1974), a widely used self‑report measure comprising Anxiety and Depression subscales. Items are rated on a 4‐point Likert scale ranging from 1 (*not at all*) to 4 (*extremely*), with higher mean subscale scores indicating higher levels of symptom severity. Mean subscale scores of 1.75 or higher were used to indicate clinically relevant symptoms (M. Jakobsen et al., 2011). The HSCL‐25 has been extensively validated in refugee populations across diverse cultural contexts (e.g., Wind et al., 2017). In the present study, internal consistency was excellent for both the Anxiety, Cronbach's α = .89, and Depression, Cronbach's α = .92, subscales.

##### Posttraumatic stress

Posttraumatic stress symptoms were assessed using the four‐item PTSD Checklist for *DSM‐5*–Short Form (PCL‐SF; Zuromski et al., 2019), which is based on the widely used 20‐item PCL‐5 (Weathers et al., 2013). Items are tied to symptoms outlined in the *Diagnostic and Statistical Manual of Mental Disorders* (5th ed.; *DSM‐5*; American Psychiatric Association, 2013) and rated on a scale of 0 (*not at all*) to 4 (*extremely*). Scores are summed to yield a total score (range: 0–16), with higher scores reflecting higher levels of symptom severity. A cutoff score of 6 or higher was used to indicate a probable clinically relevant symptom level (Forkus et al., 2023). The PCL‐SF has demonstrated psychometric properties comparable to the full PCL‐5 scale (Zuromski et al., 2019). Internal consistency in the current sample was adequate, Cronbach's α = .78.

#### Social support

Perceived social support was assessed using the Oslo Social Support Scale (OSSS‐3; Kocalevent et al., 2018), a brief, self‐report measure with demonstrated validity and reliability that is used to assess the respondent's number of close confidants, perceived interest and concern from others, and availability of practical support. Items are rated on a 4‐ or 5‐point scales and summed to yield a total score (range: 3–14), with higher scores indicating higher levels of perceived social support. Internal consistency in the present study was low, Cronbach's α = .53.

### Data analysis

All analyses were performed in *R* (Version 4.5.2) using RStudio (Version 2025.09.2). LPA was performed to identify subgroups with similar patterns of stressor exposure and mental health outcomes, using the *tidyLPA* package (Rosenberg & van Lissa, 2018). Profiles were estimated based on two indicators of stressor exposure and three indicators of mental health outcomes. Due to systematic errors in trauma exposure and HSCL‐25 items and the resulting non–missing completely at random (MCAR) missingness (J. C. Jakobsen et al., 2017), Tigrinya‐ and Pashto‐speaking participants were excluded. For the remaining data, Little's (1988) test supported the MCAR assumption, χ^2^(6,354, *N* = 437) = 917.0, *p* = 1.00.

Missing data were handled using multiple imputation (MI) with chained equations at the item level (Buuren & Groothuis‐Oudshoorn, 2011), as the proportion of missing outcome data was low (5.7%; see Supplementary Figures S1 and S2). MI is a viable alternative to full information maximum likelihood (FIML) in such cases and allows for use across models (Depaoli et al., 2025; Sinha et al., 2021). Analyses involving covariates used complete‑case data. Reporting follows the adapted GRoLTS checklist (van de Schoot et al., 2017; Supplementary Table S2).

Models with increasing profile numbers and varying variance–covariance structures were estimated sequentially, using hierarchical clustering–based start values (Wardenaar, 2021). Full model specifications and selection details are provided in the Supplementary Materials. Model fit was evaluated using multiple indices (Nylund et al., 2007), including log‐likelihood, Bayesian information criterion (BIC), sample‐size adjusted BIC (ssaBIC), Akaike information criterion (AIC), the bootstrap likelihood ratio test (BLRT), and entropy. Lower log‐likelihood, BIC, ssaBIC, and AIC values indicated better fit, whereas higher entropy values reflected greater classification accuracy; significant BLRT supported the *k* profile over the *k*‐1 profile model. Final model selection also considered interpretability and parsimony. Symptom differences between profiles were examined using bootstrapped robust analyses of variance (ANOVAs) with post hoc tests (Jongedijk et al., 2020; Wilcox, 2012) in the *WRS2* package (Mair & Wilcox, 2020).

Predictors of latent profile membership were examined using a three‑step approach (Nylund‐Gibson et al., 2019). First, latent profiles were estimated without covariates. Second, individuals were assigned to their most likely profile while retaining information on classification uncertainty. Third, a multinomial logistic regression was conducted using the *mlogit* package (Croissant, 2020).

## RESULTS

### Sample characteristics

A total of 437 FDP were included in the sample; 44.1% were female, and the mean age was 33.8 years (*SD* = 13.0). Participants predominantly originated from Ukraine (32.7%), Afghanistan (16.3%), or Türkiye (16.3%). Approximately half of the sample (48.5%) had lived in Switzerland for 1 year or less at the time of assessment and held a secure visa status (50.1%). Regarding relationship status, 42.5% were married or in a committed relationship. Educational attainment level varied considerably: 19.0% of participants had received fewer than 4 years of schooling, whereas 28.4% reported more than 12 years. Only 6.2% of participants reported paid employment, whereas 25.3% were unemployed and 33.6% were unable to work. Sociodemographic characteristics are provided in Table 1.

**TABLE 1:** Sociodemographic characteristics of the sample.

Characteristic			Included in analysis	
	*M*	*SD*	*n*	%
Age (years)	33.78	13.02	434	99.3

	***n***	**%**	***n***	**%**
Gender				
Female	192	44.1	435	99.5
Country of Origin			245	56.1
Ukraine	80	32.6		
Afghanistan	40	16.3		
Türkiye	40	16.3		
Iran	20	8.2		
Sri Lanka	6	2.4		
Syria	6	2.4		
Ethiopia	5	2.0		
Russia	5	2.0		
Others	43	17.5		
Time in Switzerland (years)			398	91.1
< 1–1	193	48.5		
2–3	170	42.7		
≥ 4	35	8.8		
Marital status				
Married/in a relationship	184	42.5	433	99.1
Not married/not in a relationship	249	57.5		
Visa status			435	99.5
Insecure	217	49.9		
Secure	218	50.1		
Education (years)			430	98.4
< 4	82	19.0		
4–8	100	23.2		
9–12	126	29.2		
≥ 12	122	28.4		
Occupation			241	55.1
Employed	15	6.2		
Unemployed	61	25.3		
Unable or not allowed to work	81	33.6		
Unpaid training	39	16.2		
Voluntary or unpaid work	20	8.3		
Retired	6	2.5		
Other	19	7.9		

*Note*: *N* = 437.

A considerable proportion of participants met clinical thresholds for probable depression, anxiety, and PTSD, indicating a high burden of co‐occurring psychopathology. Participants also reported having experienced and/or witnessed a wide range of PTE types (*M* = 3.4, *SD* = 2.0, range: 0–7), indicating considerable cumulative trauma exposure, and a high number of postmigration stressors (*M* = 4.3, *SD* = 2.4, range: 0–9), reflecting ongoing exposure to significant psychosocial stressors. Supplementary Table S3 provides further details on descriptive statistics of the outcome variables.

### Identification of latent profiles

Fit indices for the one‑ to six‑profile solutions of Model 2 are presented in Table 2 (see Supplementary Tables S4– S6 and Supplementary Figures S3–S6 for additional models). Initial analyses suggested several plausible solutions, including Model 1 with three classes, and Models 2 and 6 with four and five classes. All solutions showed significant BLRT values, indicating improved fit over models with one fewer class. However, only Models 1 and 2 demonstrated high entropy (i.e., > .85), suggesting good classification accuracy. Profile plots for Model 1 (three classes) indicated that classes primarily reflected differences in overall severity rather than qualitatively distinct patterns, consistent with the “salsa effect” (Sinha et al., 2021). Profile size was also considered (see Supplementary Table S7), with prior thresholds ranging from greater than 1% (Spurk et al., 2020) to greater than 10% (Wang et al., 2025). Both Model 2 solutions met these criteria, indicating acceptable class proportions. Within Model 2, BIC values decreased from the one‐ to the five‐profile solution, whereas AIC and ssaBIC values steadily declined up to the six‑profile solution, indicating improved fit for the five‐profile solution. Considering model fit, parsimony, classification quality, profile size, and substantive interpretability, the five‑profile solution of Model 2 was deemed most meaningful and retained as the final solution.

**TABLE 2:** Goodness of fit indices for profile solutions.

Number of profiles	Log‐likelihood	AIC	BIC	ssaBIC	Entropy	BLRT *p*
1	−3,097.88	6,215.76	6,256.56	6,224.82	1.00	
2	−2,778.00	5,598.00	5,683.67	5,617.03	.85	< .001
3	−2,664.69	5,393.38	5,523.94	5,422.38	.89	< .001
4	−2,603.35	5,292.69	5,468.13	5,331.67	.89	< .001
**5**	**−2,570.19**	**5,248.37**	**5,468.69**	**5,297.32**	**.87**	< .001
6	−2,557.39	5,244.77	5,509.97	5,303.69	.85	.078

*Note*: Bolding indicates the best‐fitting model. AIC = Akaike information criterion; BIC = Bayesian information criterion; ssaBIC = sample size–adjusted BIC; BLRT = bootstrap likelihood ratio test.

Figure 1 shows the standardized mean symptom scores of stressor exposure and mental health clusters in the five‐profile solution. Profile 1 (*n* = 49, 11.2%) was characterized by very high stressor exposure and very high symptom levels, with the highest stressor load and symptom levels for all three mental health indicators. Profile 2 (*n* = 188, 43.0%), comprising the largest group of FDP, showed high stressor exposure and high symptom levels. Profile 3 (*n* = 126, 28.8%) reflected moderate stressor exposure and moderate symptom levels. Profile 4 (*n* = 52, 11.9%) was characterized by moderate stressor exposure and low symptom levels. Profile 5 (*n* = 22, 5.0%) showed low stressor exposure and no symptoms.

**FIGURE 1:**
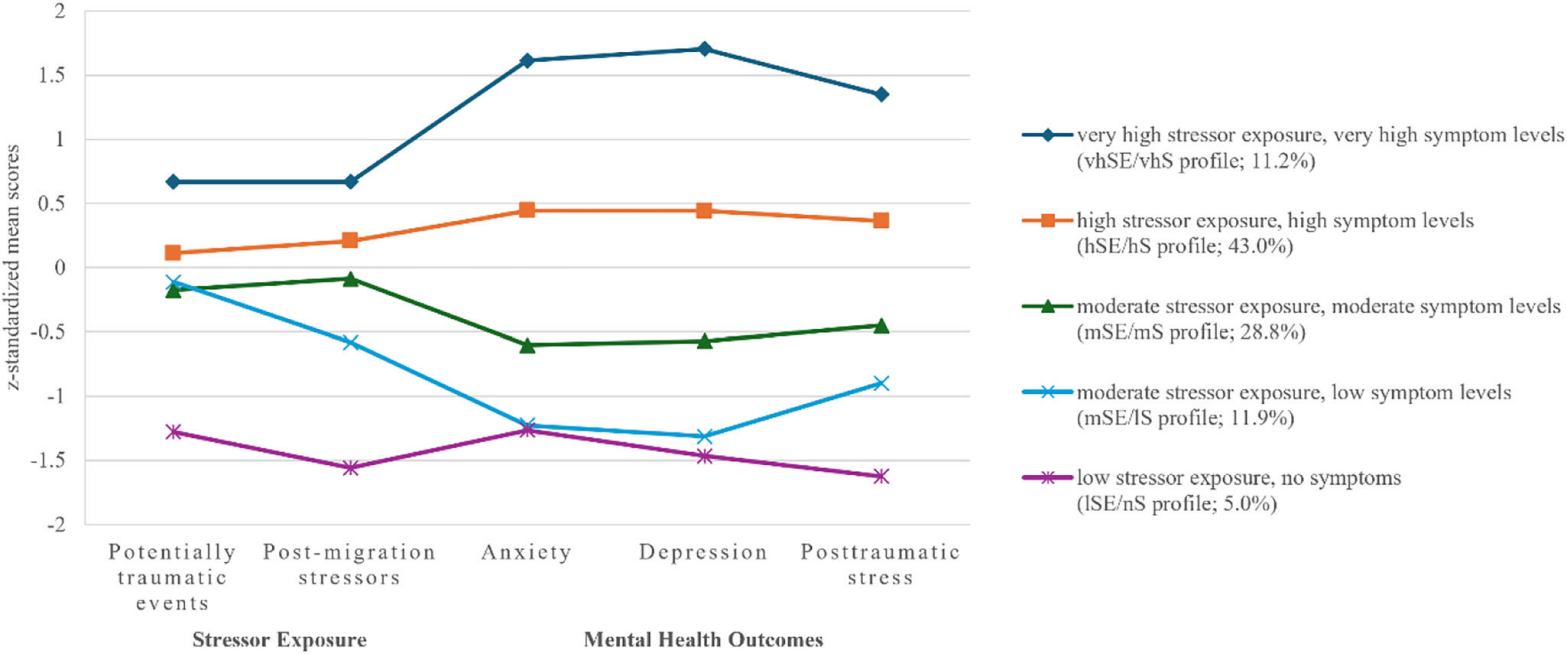
Standardized mean symptom scores of stressor exposure and mental health profiles in the five‐profile solution

Results of the robust ANOVAs with 20% trimmed means revealed significant group differences across all five outcome variables, *F_t_* s = 31.526–821.093, *p*s < .001. Post hoc comparisons indicated that regarding symptom levels (depression, anxiety, PTSD), significant differences were observed between most profiles except Profiles 4 and 5, which did not differ significantly in terms of anxiety levels. All profiles differed in postmigration stressor exposure, whereas no significant differences in PTE exposure were found between Profiles 2 and 4 or between Profiles 3 and 4. Based on these differences in overall stressor exposure and mental health symptom levels, the five profiles were labelled as (a) *very high stressor exposure, very high symptom levels* (vhSE/vhS); (b) *high stressor exposure, high symptom levels* (hSE/hS); (c) *moderate stressor exposure, moderate symptom levels* (mSE/mS); (d) *moderate stressor exposure, low symptom levels* (mSE/lS); and (e) *low stressor exposure, no symptoms* (lSE/nS). Supplementary Table S8 presents mean values of outcome variables across profiles.

### Predictors of profile membership

Descriptive statistics for predictors are shown in Supplementary Table S9. Compared to participants in the mSE/lS profile, those in the vhSE/vhS and hSE/hS profiles were more likely to be female and have an insecure visa status. Additionally, compared to participants in the mSE/lS profile, those in the vhSE/vhS profile were more likely to receive less social support and have completed 12 years or more of education. Compared to the mSE/lS profile, the mSE/mS profile included more female FDP, whereas individuals in the lSE/nS profile were more likely to have completed 12 years or more of education than those in the mSE/lS profile. Relative to participants in the lSE/nS profile, those in the vhSE/vhS profile were more likely to be female, have insecure visa status, and report lower social support. Compared to the mSE/mS profile, the vhSE/vhS profile was also associated with female gender, insecure visa status, lower social support, and higher education. Finally, compared to hSE/hS, the vhSE/vhS profile was more likely to involve insecure visa status. The results of the multinomial logistic regression model are shown in Supplementary Table S10.

## DISCUSSION

The present study is, to our knowledge, the first to identify homogenous subgroups of FDP characterized by shared patterns of stressor exposure and mental health outcomes. Latent profile analysis revealed five profiles, characterized as vhSE/vhS (11.2%), hSE/hS (43.0%), mSE/mS (28.8%), mSE/lS (11.9%), and lSE/nS (5.0%). The largest subgroup (43.0%) showed both high stressor exposure and high symptom levels.

The identification of qualitatively distinct patterns was partially supported by the identification of one subgroup (mSE/lS profile) in which participants reported lower symptom levels than other groups with similar stressor exposure levels, suggesting resilient functioning (Kalisch et al., 2015, 2021). Although symptom levels in other profiles largely corresponded to stressor exposure, this pattern of the mSE/lS profile indicates that the association between stressor exposure and mental health outcomes is not strictly linear. Further, findings from an LCA study suggested that the type of stressor exposure, rather than simply the frequency, may be particularly relevant for understanding variations in outcomes (Byrow et al., 2022). Together, these results underscore the importance of recognizing the complexity in trauma responses.

Several predictors of stressor exposure and mental health profiles were identified. First, social support and secure visa status emerged as key protective factors distinguishing high‐ and lower‐symptom profiles. Participants with lower perceived social support and insecure visa status were more likely to belong to the vhSE/vhS profile compared to the mSE/lS profile, consistent with evidence on the buffering effects of these two factors (Nickerson et al., 2024). However, when comparing profiles with similar levels of stressor exposure, social support and secure visa status no longer significantly predicted resilient functioning, suggesting that these factors influence symptom levels but not necessarily coping capacity. This may partly reflect methodological factors (e.g., differences in power related to subgroup sample sizes) or domain‐specific effects of social support, which may be more strongly linked to depressive symptoms (Price et al., 2018). As the present study only assessed perceived social support, the findings might vary when considering other aspects of social support, such as received social support (Wachter et al., 2022). An alternative explanation is that higher‐level mechanisms may play a more central role in coping with a high stressor load rather than single factors. Prior research shows that predictors of resilience often have small effect sizes, making resilient functioning difficult to forecast (Bonanno, 2021). Mechanisms such as regulatory flexibility (Bonanno et al., 2024) and positive appraisal style (Kalisch et al., 2015) may, therefore, be more critical to understanding adaptation under high stressor load. The current findings underscore the need for future research to disentangle the relative contributions of different resilience factors and mechanisms.

Gender differences were also evident, with women more likely to belong to high‐symptom profiles, even when comparing profiles with similar stressor exposure. This aligns with broader evidence indicating that women experience PTSD at 2–3 times the rate of men (Olff, 2017). Possible explanations include gender role expectations, hormonal factors, and genetic vulnerability (Christiansen & Berke, 2020). Particularly in contexts of forced displacement, women may also face unique vulnerabilities, such as gender‐based violence (Tadesse et al., 2024), caregiving responsibilities (Wachter et al., 2016), and limited access to resources (DeSa et al., 2022). Observed gender differences may also reflect differential exposure to postevent adversities and unequal access to support (Mao & Agyapong, 2021), suggesting that differences may arise less from inherent stressor sensitivity and more from exposure to distinct stressors and the use of different coping strategies (Kalisch et al., 2024). Reporting bias may further contribute, as men generally underreport mood‑related symptoms and perceive them as less severe (Shi et al., 2021).

Sociodemographic factors showed mixed effects. Educational attainment was associated with both high‐symptom profiles and profiles characterized by low stressor exposure and no symptoms, reflecting its dual role as both a protective factor (Barbui et al., 2023) and a source of distress due to loss of status and limited opportunities in displacement contexts (Nickerson et al., 2024). Interestingly, neither age, length of residency, nor relationship status predicted profile membership. Overall, these findings underscore the complexity of sociodemographic influences and highlight the need for models that integrate psychosocial and contextual factors to better explain psychological adjustment among FDP (Hynie, 2018).

We note several methodological limitations. First, outcomes were assessed exclusively through self‑report measures rather than structured clinical interviews, potentially introducing measurement bias. Although established instruments were used, some translations lacked formal psychometric validation. Second, the self‐selected sample of individuals with elevated distress enrolled in a stress‐management course may limit generalizability, highlighting the need for more representative samples. Third, PTEs unrelated to forced displacement (e.g., childhood trauma) were not assessed, which may have affected participants’ overall stressor load (Miller & Rasmussen, 2024). Fourth, stressor exposure was measured using simple counts of PTEs and postmigration stressors, an approach that assumes all stressors contribute equally to distress and may obscure differential effects (Byrow et al., 2022; Rasmussen et al., 2018). Fifth, one latent profile represented 5% of participants, raising concerns about statistical stability despite potential clinical relevance. Sixth, the internal consistency of the social support measure was low (Cronbach's α = .53), constraining interpretation and underscoring the need for more comprehensive assessment. Finally, the cross‐sectional study design precludes causal inference.

Overall, the current findings suggest that stressor exposure and mental health outcomes are not uniformly correlated among FDP, revealing substantial heterogeneity in their psychological responses. By identifying distinct profiles, this study offers a more nuanced understanding of FDP mental health that moves beyond a deficit‐based perspective to acknowledge resilience and variability in adjustment to adversity. These insights underscore the importance of tailoring interventions to individual profiles. Future research should replicate these findings across diverse FDP groups and explore longitudinal trajectories to better inform prevention and intervention strategies.

## AUTHOR NOTE

This research project was supported by the Swiss National Science Foundation (SNSF; Grant No. CRSII5_209491) and funded by Promotion Sante ´Suisse (Agreement No. 20.186.K50001). The funding sources had no role in the study design; collection, analysis, or interpretation of the data; writing of the manuscript; or decision to submit the paper for publication.

During the preparation of this work, Microsoft Copilot was used to condense the text and increase its readability. After using this tool, the authors reviewed and edited the content as needed, and we take full responsibility for the content of the publication.

We sincerely thank all participants for taking part in this study and are grateful to our assessors for their support during the assessment process. We also thank our partner organizations for their support with study recruitment and coordination of assessments, as well as Beratungen und Analysen (B&A) for their assistance with data curation.

## OPEN PRACTICES STATEMENT

This study was not formally preregistered. Data will be shared publicly once data collection of the entire study has been completed.

## AUTHOR CONTRIBUTIONS

**Lisa Dasen**: conceptualization, methodology, formal analysis, supervision, project administration, writing–original draft, writing–review and editing. **Jennifer Kurath**: writing–review and editing, supervision. **Béatrice Strock**: writing–review and editing, supervision. **Karl Juri Weinreich**: writing–review and editing. **Julia Spaaij**: writing–review and editing, funding acquisition. **Matthis Schick**: funding acquisition, writing–review and editing. **Angela Nickerson**: writing–review and editing, conceptualization. **Richard A. Bryant**: writing–review and editing, supervision, funding acquisition, conceptualization. **Naser Morina**: conceptualization, writing–review and editing, funding acquisition, supervision.

## Supporting information



SUPPORTING INFORMATION
