# Supplementary material for: Exploring stressor exposure and mental health symptom patterns among forcibly displaced people: A latent profile analysis
Source: J Trauma Stress. 2026 Jul 28;39(5):950–61. doi: 10.1002/jts.70110 (PMC13639206; doi:10.1002/jts.70110)
Supplement: Supplementary file 1 [file JTS-39-950-s001.docx]

**Appendix A. Supplementary material**

**Table S1. A full list of items used to measure Stressor Exposure.**

| **Potentially traumatic events** | **Post-migration stressors** |
| --- | --- |
| War or other conflict | Communication difficulties |
| Violence (physical or sexual) | Discrimination |
| Imprisonment or kidnapping | Worry about family members |
| Political or religious persecution | Employment |
| Natural disaster (e.g. flood or drought) | Immigration processes |
| Other traumatic event | Access to health and welfare services |
|  | Adjustment to life in Switzerland |
|  | Transport |
|  | Loneliness, boredom, or isolation |

**Table S2**. Adapted Guidelines for Reporting on Latent Trajectory Studies Checklist (Petersen et al., 2019)

| **Checklist Item** | **Reported?** |
| --- | --- |
| Is the missing data mechanism reported? | Yes |
| Is a description provided of what variables are related to attrition/ missing data? | Yes |
| Is a description provided of how missing data in the analyses were dealt with? | Yes |
| Is information about the distribution of the observed variables included? e.g., continuous, ordinal, count, zero inflated | Yes |
| Is the software mentioned? | Yes |
| Are parameter restrictions reported? | Yes |
| If covariates have been used, can analyses still be replicated? i.e., is sufficient detail provided about the method used to include covariates? | Yes |
| Is information reported about the number of random start values and final iterations included? | Yes |
| Are the model comparison (and selection) tools described from a statistical perspective? | Yes |
| Are the total number of fitted models reported, including a one-class solution? | Yes |
| Are the number of cases per class reported for each profile solution (absolute sample size, or proportion)? | Yes |
| Is entropy reported? | Yes |
| Are plots/barcharts included with the response patterns of the classes/profiles in the final solution? | Yes |
| Are plots/barcharts included with the response patterns of the classes/profiles for each model? | Yes |
| Are characteristics of the final class solution numerically described? (i.e., means, SD/SE, n, CI, etc.) | Yes |
| Are the syntax files available (either in the appendix, supplementary materials, or from the authors)? | Yes |

**Figure S1.** Percentage of Missing Outcome Data

**Figure S2.** Number of Missing Data for individual variables

**Table S3.** Descriptive statistics, Cronbach's Alpha and correlations between outcome variables.

|  | Total  (N = 437) | | % above clinical thresholds | | α | Depression | PTSD | PTE | PMLD | Social Support |
| --- | --- | --- | --- | --- | --- | --- | --- | --- | --- | --- |
|  | *M* | *SD* | n | % |  |  |  |  |  |  |
| Distress load |  |  |  |  |  |  |  |  |  |  |
| Anxiety | 2.19 | 0.7 | 292 | 66.82 | .90 | .81*** | .60*** | .29*** | .30*** | -.18*** |
| Depression | 2.35 | 0.71 | 337 | 77.12 | .92 |  | .68*** | .27*** | .40*** | -.22*** |
| PTSD | 7.03 | 4.14 | 264 | 60.41 | .78 |  |  | .31*** | .44*** | -.11* |
| Stressor load |  |  |  |  |  |  |  |  |  |  |
| PTE | 3.35 | 2.04 |  |  | .72 |  |  |  | .18*** | -.07 |
| PMLD | 4.32 | 2.36 |  |  | .71 |  |  |  |  | -.14* |
| Social Support | 7.72 | 2.35 |  |  | .54 |  |  |  |  |  |

*Note.* *** p < .001, * p < .005, α = Cronbach's Alpha, PTSD = Posttraumatic Stress Disorder, PTE = Potentially traumatic events, PMLD = Postmigration living difficulties.

**Table S4.** Fit indices for model 1 (equal variances, and covariances fixed to 0)

| **Model** | **Classes** | **Log-Likelihood** | **AIC** | **BIC** | **SABIC** | **Entropy** | **BLRT p value** |
| --- | --- | --- | --- | --- | --- | --- | --- |
| 1 | 1 | -3097.88 | 6215.76 | 6256.56 | 6224.82 | 1.00 |  |
| 1 | 2 | -2804.40 | 5640.80 | 5706.08 | 5655.30 | 0.82 | 0.00 |
| 1 | 3 | -2689.56 | 5423.11 | 5512.87 | 5443.05 | 0.86 | 0.00 |
| 1 | 4 | -2649.15 | 5354.30 | 5468.54 | 5379.68 | 0.81 | 0.00 |
| 1 | 5 | -2630.76 | 5329.52 | 5468.24 | 5360.34 | 0.79 | 0.00 |
| 1 | 6 | -2618.10 | 5316.20 | 5479.39 | 5352.45 | 0.75 | 0.00 |

*Note.* AIC = Akaike information criterion; BIC = Bayesian information criterion; saBIC = simple-size adjusted BIC; BLRT = Bootstrap likelihood ratio test.

**Table S5.** Fit indices for model 3 (equal variances and equal covariances)

| **Model** | **Classes** | **Log-Likelihood** | **AIC** | **BIC** | **SABIC** | **Entropy** | **BLRT p value** |
| --- | --- | --- | --- | --- | --- | --- | --- |
| 3 | 1 | -2645.01 | 5330.02 | 5411.61 | 5348.14 | 1.00 |  |
| 3 | 2 | -2624.64 | 5301.28 | 5407.36 | 5324.85 | 0.71 | 0.00 |
| 3 | 3 | -2603.66 | 5271.32 | 5401.88 | 5300.33 | 0.84 | 0.29 |
| 3 | 4 | -2598.02 | 5272.05 | 5427.08 | 5306.49 | 0.76 | 0.00 |
| 3 | 5 | -2591.87 | 5271.74 | 5451.25 | 5311.62 | 0.76 | 0.67 |
| 3 | 6 | -2575.70 | 5251.40 | 5455.40 | 5296.73 | 0.79 | 0.04 |

*Note.* AIC = Akaike information criterion; BIC = Bayesian information criterion; saBIC = simple-size adjusted BIC; BLRT = Bootstrap likelihood ratio test.

**Table S6.** Fit indices for model 6 (varying variances and varying covariances)

| **Model** | **Classes** | **Log-Likelihood** | **AIC** | **BIC** | **SABIC** | **Entropy** | **BLRT p value** |
| --- | --- | --- | --- | --- | --- | --- | --- |
| 6 | 1 | -2645.01 | 5330.02 | 5411.61 | 5348.14 | 1.00 |  |
| 6 | 2 | -2593.46 | 5268.92 | 5436.20 | 5306.08 | 0.75 | 0.00 |
| 6 | 3 | -2552.95 | 5229.91 | 5482.86 | 5286.11 | 0.75 | 0.01 |
| 6 | 4 | -2522.94 | 5211.88 | 5550.51 | 5287.11 | 0.81 | 0.00 |
| 6 | 5 | -2490.30 | 5188.60 | 5612.92 | 5282.87 | 0.83 | 0.04 |
| 6 | 6 |  |  |  |  |  |  |

*Note.* AIC = Akaike information criterion; BIC = Bayesian information criterion; saBIC = simple-size adjusted BIC; BLRT = Bootstrap likelihood ratio test.

**Figure S3.** Profile plots for model 1

**Figure S4.** Profile plots for model 2

**Figure S5.** Profile plots for model 3

**Figure S6.** Profile plots for model 6

**Table S7.** Numbers of participants in each profile for each profile solution of model 2

|  | | Number of profiles | | | | | | | | | | | |
| --- | --- | --- | --- | --- | --- | --- | --- | --- | --- | --- | --- | --- | --- |
|  | 1 | | | 2 | | 3 | | 4 | | 5 | | 6 | |
|  | *n* | | % | *n* | % | *n* | % | *n* | % | *n* | % | *n* | % |
| 2-profile solution | 180 | | 41.2 | 257 | 58.8 |  |  |  |  |  |  |  |  |
| 3-profile solution | 152 | | 34.8 | 232 | 53.1 | 53 | 12.1 |  |  |  |  |  |  |
| 4-profile solution | 148 | | 33.9 | 209 | 47.8 | 29 | 6.64 | 51 | 11.7 |  |  |  |  |
| 5-profile solution | 22 | | 5.0 | 52 | 11.9 | 126 | 28.8 | 188 | 43.0 | 49 | 11.2 |  |  |
| 6-profile solution | 22 | | 5.0 | 55 | 12.6 | 70 | 16.0 | 55 | 12.6 | 186 | 42.6 | 49 | 11.2 |

*Note.* n = number

**Table S8.** Means of outcome variables across profiles

|  | very high stressor exposure, very high symptom profile  (vhSE/vhS profile; n = 49, 11.2%) | | high stressor exposure, high symptom profile  (hSE/hS profile; n = 188, 43.0%) | | moderate stressor exposure, moderate symptom profile  (mSE/mS profile; n = 126, 28.8%) | | moderate stressor exposure, low symptom profile  (mSE/lS profile; n = 52, 11.9%) | | low stressor exposure, no symptom profile  (lSE/nS profile; n = 22, 5.0%) | |  |  |
| --- | --- | --- | --- | --- | --- | --- | --- | --- | --- | --- | --- | --- |
|  | *M* | *SD* | *M* | *SD* | *M* | *SD* | *M* | *SD* | *M* | *SD* | *F* | *ξ* |
| Distress load |  |  |  |  |  |  |  |  |  |  |  |  |
| Anxiety | 3.33 | 0.33 | 2.51 | 0.43 | 1.77 | 0.27 | 1.33^a^ | 0.20 | 1.30^a^ | 0.28 | 330.6552 | 0.85 |
| Depression | 3.57 | 0.19 | 2.69 | 0.35 | 1.95 | 0.27 | 1.42 | 0.21 | 1.31 | 0.27 | 821.0933 | 0.83 |
| PTSD | 12.69 | 2.56 | 8.56 | 3.17 | 5.26 | 2.82 | 3.23 | 1.59 | 0.32 | 0.48 | 370.9664 | 0.81 |
| Stressor load |  |  |  |  |  |  |  |  |  |  |  |  |
| PTE | 4.73 | 1.71 | 3.59^a^ | 1.99 | 3.01^b^ | 1.82 | 3.08^ab^ | 2.13 | 0.77 | 0.81 | 31.5259 | 0.61 |
| PMLD | 5.94 | 2.01 | 4.8 | 2.1 | 4.19 | 2.25 | 2.94 | 2.0 | 0.68 | 0.72 | 95.3675 | 0.68 |
| Participants above clinical thresholds | *n* | *%* | *n* | *%* | *n* | *%* | *n* | *%* | *n* | *%* |  |  |
| Anxiety | 49 | 100.0 | 179 | 95.21 | 62 | 49.21 | 1 | 1.92 | 1 | 4.55 |  |  |
| Depression | 49 | 100.0 | 188 | 100.0 | 97 | 76.98 | 1 | 1.92 | 2 | 9.09 |  |  |
| PTSD | 49 | 100.0 | 159 | 84.57 | 52 | 41.27 | 4 | 7.69 | 0 | 0 |  |  |

*Note.* ^***^*p* < .001. Post hoc tests: Mean values with equal letters (a, b) are not significantly different (p > .05). PTSD = Posttraumatic Stress Disorder, PTE = Potentially traumatic events, PMLD = Post-migration living difficulties.

**Table S9.** Descriptive statistics of the predictor variables within each of the six latent profiles.

|  | very high stressor exposure, very high symptom profile  (vhSE/vhS profile; n = 49, 11.2%) | | high stressor exposure, high symptom profile  (hSE/hS profile; n = 188, 43.0%) | | moderate stressor exposure, moderate symptom profile  (mSE/mS profile; n = 126, 28.8%) | | moderate stressor exposure, low symptom profile  (mSE/lS profile; n = 52, 11.9%) | | low stressor exposure, no symptom profile  (lSE/nS profile; n = 22, 5.0%) | |
| --- | --- | --- | --- | --- | --- | --- | --- | --- | --- | --- |
|  | N | % | N | % | N | % | N | % | N | % |
| Gender |  |  |  |  |  |  |  |  |  |  |
| female | 27 | 55.10 | 87 | 46.77 | 56 | 44.44 | 16 | 30.77 | 6 | 27.27 |
| male | 22 | 44.90 | 99 | 53.23 | 70 | 55.56 | 36 | 69.23 | 16 | 72.73 |
| Visa status |  |  |  |  |  |  |  |  |  |  |
| insecure | 39 | 79.59 | 93 | 49.47 | 55 | 43.65 | 18 | 36.0 | 12 | 54.55 |
| secure | 10 | 20.41 | 95 | 50.53 | 71 | 56.35 | 32 | 64.0 | 10 | 45.45 |
| Years in Switzerland |  |  |  |  |  |  |  |  |  |  |
| <1 - 1 | 20 | 46.51 | 77 | 45.56 | 57 | 48.31 | 28 | 59.57 | 11 | 52.38 |
| 2 - 3 | 19 | 44.19 | 75 | 10.06 | 50 | 42.37 | 16 | 34.04 | 10 | 47.62 |
| ≥4 | 4 | 9.30 | 17 | 44.38 | 11 | 9.32 | 3 | 6.38 | 0 | 0 |
| Marital status |  |  |  |  |  |  |  |  |  |  |
| Married / in a relationship | 21 | 42.86 | 76 | 40.86 | 57 | 45.24 | 23 | 46.00 | 7 | 31.82 |
| Not married /  not in a relationship | 28 | 57.14 | 110 | 59.14 | 69 | 54.76 | 27 | 54.00 | 15 | 68.18 |
| Education |  |  |  |  |  |  |  |  |  |  |
| < 4 | 7 | 14.58 | 33 | 17.74 | 23 | 18.55 | 16 | 32.0 | 3 | 13.64 |
| 4-8 | 9 | 18.75 | 41 | 22.04 | 36 | 29.03 | 9 | 18.0 | 5 | 20.73 |
| 9-12 | 12 | 25.0 | 60 | 32.26 | 32 | 25.81 | 17 | 34.0 | 5 | 22.73 |
| 12+ | 20 | 41.67 | 52 | 27.96 | 33 | 26.61 | 8 | 16.0 | 9 | 40.91 |
|  | ***M*** | ***SD*** | ***M*** | ***SD*** | ***M*** | ***SD*** | ***M*** | ***SD*** | ***M*** | ***SD*** |
| Age, years (M, SD) | 33.96 | 11.89 | 32.96 | 12.29 | 35.23 | 13.82 | 32.62 | 12.69 | 34.68 | 17.33 |
| Social Support (M, SD) | 6.59 | 2.23 | 7.56 | 2.32 | 8.14 | 2.24 | 8.15 | 2.34 | 8.18 | 2.79 |

*Note.* M = mean, N = number, SD = standard deviation.

**Table S10.** Results of the multinomial regression analyses of the five profiles

|  | very high stressor exposure, very high symptom profile (vhSE/vhS profile) | | | | | | high stressor exposure, high symptom profile (hSE/hS profile) | | | | | | moderate stressor exposure, moderate symptom profile (mSE/mS profile) | | | | | | low stressor exposure, no symptom profile (lSE/nS profile) | | | | | |
| --- | --- | --- | --- | --- | --- | --- | --- | --- | --- | --- | --- | --- | --- | --- | --- | --- | --- | --- | --- | --- | --- | --- | --- | --- |
|  | B | SE | p | OR | 95% CI | | B | SE | p | OR | 95% CI | | B | SE | p | OR | 95% CI | | B | SE | p | OR | 95% CI | |
|  |  |  |  |  | *LL* | *UL* |  |  |  |  | *LL* | *UL* |  |  |  |  | *LL* | *UL* |  |  |  |  | *LL* | *UL* |
| vs. moderate stressor exposure, low symptom profile (mSE/lS profile) | | | | | | |  |  |  |  |  |  |  |  |  |  |  |  |  |  |  |  |  |  |
| Gender |  |  |  |  |  |  |  |  |  |  |  |  |  |  |  |  |  |  |  |  |  |  |  |  |
| Female | 2.029*** | .541 | <.001 | 7.605 | 2.632 | 21.97 | 1.242** | .416 | .003 | 3.461 | 1.531 | 7.823 | 1.019* | 0.426 | 0.017 | 2.769 | 1.201 | 6.386 | 0.251 | 0.674 | 0.710 | 1.285 | 0.343 | 4.815 |
| Age | .009 | .020 | .667 | 1.009 | 0.969 | 1.05 | 0.003 | .016 | .871 | 1.003 | 0.973 | 1.033 | 0.012 | 0.016 | 0.454 | 1.012 | 0.981 | 1.043 | 0.025 | 0.023 | 0.274 | 1.025 | 0.981 | 1.071 |
| Visa status | |  |  |  |  |  |  |  |  |  |  |  |  |  |  |  |  |  |  |  |  |  |  |  |
| Secure | -2.602*** | .544 | <.001 | .074 | 0.026 | 0.215 | -1.022** | .383 | .008 | 0.36 | 0.17 | 0.763 | -0.757+ | 0.395 | 0.055 | 0.469 | 0.216 | 1.018 | -1.046+ | 0.596 | 0.079 | 0.351 | 0.109 | 1.129 |
| Marital status | |  |  |  |  |  |  |  |  |  |  |  |  |  |  |  |  |  |  |  |  |  |  |  |
| married | -.216 | .514 | .675 | .806 | 0.294 | 2.208 | -.409 | .391 | .296 | 0.664 | 0.308 | 1.43 | -0.236 | 0.400 | 0.556 | 0.79 | 0.36 | 1.731 | -0.742 | 0.601 | 0.217 | 0.351 | 0.109 | 1.129 |
| Length of stay | |  |  |  |  |  |  |  |  |  |  |  |  |  |  |  |  |  |  |  |  |  |  |  |
| 2-3 | .428 | .494 | .387 | 1.534 | 0.582 | 4.045 | 0.435 | .377 | .248 | 1.546 | 0.738 | 3.236 | 0.188 | 0.390 | 0.630 | 1.207 | 0.562 | 2.592 | 0.385 | 0.562 | 0.493 | 1.47 | 0.488 | 4.427 |
| ≥4 | 1.078 | .903 | .233 | 2.939 | 0.5 | 17.26 | 0.895 | .694 | .197 | 2.447 | 0.627 | 9.546 | 0.597 | 0.717 | 0.405 | 1.817 | 0.446 | 7.408 | -15.547 | 2352.152 | 0.995 | 0 | 0 | Inf |
| Social Support | -.341** | .106 | .001 | .711 | 0.577 | 0.876 | -0.129 | .081 | .111 | 0.879 | 0.75 | 1.03 | -0.012 | 0.083 | 0.887 | 0.988 | 0.84 | 1.163 | -0.056 | 0.120 | 0.640 | 0.945 | 0.747 | 1.197 |
| Education | |  |  |  |  |  |  |  |  |  |  |  |  |  |  |  |  |  |  |  |  |  |  |  |
| 4-8 | 1.556* | .749 | .038 | 4.742 | 1.093 | 20.564 | 0.808 | .525 | .124 | 2.244 | 0.801 | 6.284 | 1.045+ | 0.536 | 0.051 | 2.842 | 0.995 | 8.119 | 1.045 | 0.867 | 0.228 | 2.842 | 0.52 | 15.545 |
| 9-12 | .949 | .689 | .168 | 2.584 | 0.67 | 9.97 | 0.327 | .464 | .481 | 1.387 | 0.559 | 3.44 | 0.130 | 0.486 | 0.788 | 1.139 | 0.44 | 2.951 | 0.461 | 0.838 | 0.583 | 1.585 | 0.307 | 8.196 |
| 12+ | 2.135** | .732 | .004 | 8.453 | 2.014 | 35.486 | 1.060+ | .548 | .053 | 2.886 | 0.985 | 8.457 | 0.940+ | 0.564 | 0.095 | 2.56 | 0.848 | 7.726 | 1.669* | 0.843 | 0.048 | 5.307 | 1.017 | 27.697 |

*Continued.*

**Table S10.** (Continued.)

|  | very high stressor exposure, very high symptom profile (vhSE/vhS profile) | | | | | | high stressor exposure, high symptom profile (hSE/hS profile) | | | | | | moderate stressor exposure, moderate symptom profile (mSE/mS profile) | | | | | | low stressor exposure, no symptom profile (lSE/nS profile) | | | | | |
| --- | --- | --- | --- | --- | --- | --- | --- | --- | --- | --- | --- | --- | --- | --- | --- | --- | --- | --- | --- | --- | --- | --- | --- | --- |
|  | B | SE | p | OR | 95% CI | | B | SE | p | OR | 95% CI | | B | SE | p | OR | 95% CI | | B | SE | p | OR | 95% CI | |
|  |  |  |  |  | *LL* | *UL* |  |  |  |  | *LL* | *UL* |  |  |  |  | *LL* | *UL* |  |  |  |  | *LL* | *UL* |
| vs. low stressor exposure, no symptom profile  (lSE/nS profile) | | | | | | |  |  |  |  |  |  |  |  |  |  |  |  |  |  |  |  |  |  |
| Gender |  |  |  |  |  |  |  |  |  |  |  |  |  |  |  |  |  |  |  |  |  |  |  |  |
| Female | 1.778** | .677 | .009 | 5.917 | 1.571 | 22.281 | 0.991+ | .592 | 0.094 | 2.693 | 0.844 | 8.587 | 0.768 | 0.601 | 0.201 | 2.154 | 0.664 | 6.991 |  |  |  |  |  |  |
| Age | -.016 | .023 | .493 | .984 | 0.941 | 1.03 | -0.022 | .019 | 0.250 | 0.978 | 0.942 | 1.016 | -0.013 | 0.019 | 0.504 | 0.987 | 0.95 | 1.025 |  |  |  |  |  |  |
| Visa status | |  |  |  |  |  |  |  |  |  |  |  |  |  |  |  |  |  |  |  |  |  |  |  |
| Secure | -1.556* | .645 | .016 | .211 | 0.06 | 0.746 | 0.024 | .524 | 0.964 | 1.024 | 0.367 | 2.859 | 0.289 | 0.534 | 0.588 | 1.335 | 0.469 | 3.804 |  |  |  |  |  |  |
| Marital status | |  |  |  |  |  |  |  |  |  |  |  |  |  |  |  |  |  |  |  |  |  |  |  |
| married | .526 | .612 | .390 | 1.692 | 0.51 | 5.614 | 0.332 | .521 | 0.524 | 1.394 | 0.502 | 3.872 | 0.506 | 0.528 | 0.338 | 1.658 | 0.589 | 4.665 |  |  |  |  |  |  |
| Length of stay | |  |  |  |  |  |  |  |  |  |  |  |  |  |  |  |  |  |  |  |  |  |  |  |
| 2-3 | .043 | .572 | .940 | 1.044 | 0.34 | 3.2 | 0.050 | .483 | 0.918 | 1.051 | 0.408 | 2.71 | -0.197 | 0.495 | 0.690 | 0.821 | 0.311 | 2.165 |  |  |  |  |  |  |
| ≥4 | 16.625 | 2352.152 | .994 | 16606334 | 0 | Inf | 16.442 | 2352.152 | 0.994 | 13829352 | 0 | Inf | 16.144 | 2352.152 | 0.995 | 10265465 | 0 | Inf |  |  |  |  |  |  |
| Social Support | -.285* | .121 | .019 | .752 | 0.593 | 0.954 | -0.073 | .102 | 0.477 | 0.93 | 0.761 | 1.136 | 0.044 | 0.105 | 0.671 | 1.045 | 0.852 | 1.283 |  |  |  |  |  |  |
| Education | |  |  |  |  |  |  |  |  |  |  |  |  |  |  |  |  |  |  |  |  |  |  |  |
| 4-8 | .512 | .939 | .586 | 1.668 | 0.265 | 10.504 | -0.236 | .781 | 0.762 | 0.789 | 0.171 | 3.65 | 0.000 | 0.791 | 1.000 | 1 | 0.212 | 4.712 |  |  |  |  |  |  |
| 9-12 | .489 | .925 | .597 | 1.63 | 0.266 | 9.991 | -0.134 | .780 | 0.864 | 0.875 | 0.19 | 4.036 | -0.330 | 0.798 | 0.679 | 0.719 | 0.15 | 3.433 |  |  |  |  |  |  |
| 12+ | .466 | .873 | .594 | 1.593 | 0.288 | 8.808 | -0.609 | .736 | 0.408 | 0.544 | 0.129 | 2.301 | -0.729 | 0.752 | 0.332 | 0.482 | 0.11 | 2.107 |  |  |  |  |  |  |

*Continued.*

**Table S10.** (Continued.)

|  | very high stressor exposure, very high symptom profile (vhSE/vhS profile) | | | | | | high stressor exposure, high symptom profile (hSE/hS profile) | | | | | | moderate stressor exposure, moderate symptom profile (mSE/mS profile) | | | | | | low stressor exposure, no symptom profile (lSE/nS profile) | | | | | |
| --- | --- | --- | --- | --- | --- | --- | --- | --- | --- | --- | --- | --- | --- | --- | --- | --- | --- | --- | --- | --- | --- | --- | --- | --- |
|  | B | SE | p | OR | 95% CI | | B | SE | p | OR | 95% CI | | B | SE | p | OR | 95% CI | | B | SE | p | OR | 95% CI | |
|  |  |  |  |  | *LL* | *UL* |  |  |  |  | *LL* | *UL* |  |  |  |  | *LL* | *UL* |  |  |  |  | *LL* | *UL* |
| Vs. moderate stressor exposure, moderate symptom profile (mSE/mS profile) | | | | | | |  |  |  |  |  |  |  |  |  |  |  |  |  |  |  |  |  |  |
| Gender |  |  |  |  |  |  |  |  |  |  |  |  |  |  |  |  |  |  |  |  |  |  |  |  |
| Female | 1.010* | .435 | .020 | 2.746 | 1.17 | 6.448 | 0.223 | .281 | 0.428 | 1.25 | 0.72 | 2.17 |  |  |  |  |  |  |  |  |  |  |  |  |
| Age | -.003 | .017 | .859 | .997 | 0.965 | 1.03 | -0.009 | .010 | 0.369 | 0.991 | 0.971 | 1.011 |  |  |  |  |  |  |  |  |  |  |  |  |
| Visa status | |  |  |  |  |  |  |  |  |  |  |  |  |  |  |  |  |  |  |  |  |  |  |  |
| Secure | -1.846*** | .462 | <.001 | .158 | 0.064 | 0.391 | -0.265 | .274 | 0.333 | 0.767 | 0.448 | 1.313 |  |  |  |  |  |  |  |  |  |  |  |  |
| Marital status | |  |  |  |  |  | -0.173 | .264 | 0.511 |  |  |  |  |  |  |  |  |  |  |  |  |  |  |  |
| married | .020 | .418 | .962 | 1.02 | 0.449 | 2.317 | 0.442 | .500 | -0.569 | 0.841 | 0.502 | 1.41 |  |  |  |  |  |  |  |  |  |  |  |  |
| Length of stay | |  |  |  |  |  |  |  |  |  |  |  |  |  |  |  |  |  |  |  |  |  |  |  |
| 2-3 | .240 | .409 | .557 | 1.272 | 0.571 | 2.833 | 0.247 | .266 | 0.353 | 1.281 | 0.76 | 2.158 |  |  |  |  |  |  |  |  |  |  |  |  |
| ≥4 | .481 | .712 | .499 | 1.618 | 0.401 | 6.525 | 0.298 | .442 | 0.500 | 1.347 | 0.566 | 3.206 |  |  |  |  |  |  |  |  |  |  |  |  |
| Social Support | -.330*** | .087 | <.001 | .719 | 0.606 | 0.853 | -0.117* | .055 | 0.032 | 0.89 | 0.799 | 0.99 |  |  |  |  |  |  |  |  |  |  |  |  |
| Education | |  |  |  |  |  |  |  |  |  |  |  |  |  |  |  |  |  |  |  |  |  |  |  |
| 4-8 | .512 | .645 | .428 | 1.668 | 0.471 | 5.909 | -0.237 | .381 | 0.535 | 0.789 | 0.374 | 1.666 |  |  |  |  |  |  |  |  |  |  |  |  |
| 9-12 | .819 | .624 | .189 | 2.268 | 0.668 | 7.701 | 0.197 | .376 | 0.601 | 1.217 | 0.583 | 2.542 |  |  |  |  |  |  |  |  |  |  |  |  |
| 12+ | 1.195* | .607 | .049 | 3.302 | 1.005 | 10.848 | 0.120 | .384 | 0.754 | 1.128 | 0.532 | 2.391 |  |  |  |  |  |  |  |  |  |  |  |  |

*Continued.*

**Table S10.** (Continued.)

|  | very high stressor exposure, very high symptom profile (vhSE/vhS profile) | | | | | | high stressor exposure, high symptom profile (hSE/hS profile) | | | | | | moderate stressor exposure, moderate symptom profile (mSE/mS profile) | | | | | | low stressor exposure, no symptom profile (lSE/nS profile) | | | | | |
| --- | --- | --- | --- | --- | --- | --- | --- | --- | --- | --- | --- | --- | --- | --- | --- | --- | --- | --- | --- | --- | --- | --- | --- | --- |
|  | B | SE | p | OR | 95% CI | | B | SE | p | OR | 95% CI | | B | SE | p | OR | 95% CI | | B | SE | p | OR | 95% CI | |
|  |  |  |  |  | *LL* | *UL* |  |  |  |  | *LL* | *UL* |  |  |  |  | *LL* | *UL* |  |  |  |  | *LL* | *UL* |
| vs. high stressor exposure, high symptom profile  (hSE/hS profile) | | | | | | |  |  |  |  |  |  |  |  |  |  |  |  |  |  |  |  |  |  |
| Gender |  |  |  |  |  |  |  |  |  |  |  |  |  |  |  |  |  |  |  |  |  |  |  |  |
| Female | .787+ | .411 | .055 | 2.197 | 0.982 | 4.914 |  |  |  |  |  |  |  |  |  |  |  |  |  |  |  |  |  |  |
| Age | .006 | .016 | .693 | 1.006 | 0.975 | 1.038 |  |  |  |  |  |  |  |  |  |  |  |  |  |  |  |  |  |  |
| Visa status | |  |  |  |  |  |  |  |  |  |  |  |  |  |  |  |  |  |  |  |  |  |  |  |
| Secure | -1.580*** | .441 | <.001 | .206 | 0.087 | 0.488 |  |  |  |  |  |  |  |  |  |  |  |  |  |  |  |  |  |  |
| Marital status | |  |  |  |  |  |  |  |  |  |  |  |  |  |  |  |  |  |  |  |  |  |  |  |
| married | .193 | .399 | .628 | 1.213 | 0.555 | 2.654 |  |  |  |  |  |  |  |  |  |  |  |  |  |  |  |  |  |  |
| Length of stay | |  |  |  |  |  |  |  |  |  |  |  |  |  |  |  |  |  |  |  |  |  |  |  |
| 2-3 | -.007 | .385 | .985 | 0.993 | 0.466 | 2.113 |  |  |  |  |  |  |  |  |  |  |  |  |  |  |  |  |  |  |
| ≥4 | .183 | .674 | .786 | 1.201 | 0.32 | 4.501 |  |  |  |  |  |  |  |  |  |  |  |  |  |  |  |  |  |  |
| Social Support | -0.213** | 0.082 | 0.010 | 0.809 | 0.688 | 0.95 |  |  |  |  |  |  |  |  |  |  |  |  |  |  |  |  |  |  |
| Education | |  |  |  |  |  |  |  |  |  |  |  |  |  |  |  |  |  |  |  |  |  |  |  |
| 4-8 | .748 | .621 | .228 | 2.114 | 0.626 | 7.141 |  |  |  |  |  |  |  |  |  |  |  |  |  |  |  |  |  |  |
| 9-12 | .622 | .589 | .290 | 1.863 | 0.588 | 5.908 |  |  |  |  |  |  |  |  |  |  |  |  |  |  |  |  |  |  |
| 12+ | 1.075+ | .572 | .060 | 2.929 | 0.954 | 8.988 |  |  |  |  |  |  |  |  |  |  |  |  |  |  |  |  |  |  |

*Note.* ^***^*p* < .001; ^**^*p* < .01, ^*^ *p* < .05; SE = standard error; OR = odds ratio; CI = confidence interval; *LL* = lower limit; *UL* = upper limit.
